# The aftermath of surviving a sudden cardiac arrest for young exercisers - a qualitative study in Norway

**DOI:** 10.1186/s12913-022-08674-z

**Published:** 2022-11-30

**Authors:** Camilla Hardeland, Ann-Chatrin Linqvist Leonardsen, Cecilie Benedicte Isern, Hilde Moseby Berge

**Affiliations:** 1 Division of Prehospital Services, Oslo University Hospital, P.O. box 4956 Nydalen, N-0424 Oslo, Norway; 2 Department of Health, Welfare and Organization, Ostfold University College, P.O. box 700, NO-1757 Halden, Norway; 3 Department of Anesthesia, Ostfold Hospital Trust, Gralum, P.O. box 300, NO-1714 Sarpsborg, Norway; 4 Faculty of Medicine, Institute of Clinical Medicine, University of Oslo, P.O box 1171 Blindern, NO-0318 Oslo, Norway; 5 Department of Sports Medicine, Oslo Sports Trauma Research Center, Norwegian School of Sport Sciences, P.O. box 4014 Ullevål Stadion, NO-0806 Oslo, Norway; 6 General Practice Research Unit, Institute of Health and Society, University of Oslo, P.O box 1130 Blindern, NO-0318 Oslo, Norway

**Keywords:** Sudden cardiac arrest, Survivors, Exercisers, Experiences, Qualitative methods

## Abstract

**Background:**

When surviving a sudden cardiac arrest (SCA), physical, cognitive, and emotional effects of surviving may be present for months or years. The survivors' family and colleagues are also highly affected by the incident. There is little knowledge about experiences of surviving SCA in individuals who prior to the incident were young and reported to exercise regularly. Consequently, the aim of this study was to explore the aftermath of surviving a SCA in young, regular exercisers.

**Methods:**

The study had a qualitative design, conducting in-depth individual interviews with SCA survivors < 50 years of age reporting to exercise ≥ 5 h/week and/or who suffered SCA during or less than 60 min after exercise. The data were analysed using systematic text condensation in-line with recommendations from Malterud.

**Results:**

18 of 31 eligible participants were included in the study. Through analysis we identified ‘Establishing a new everyday life’ as superordinate category, with subordinate categories a) being part of my surroundings, b) expecting normality but facing a new reality and c) lucky to be alive!

**Conclusion:**

This study adds knowledge about young and regular exercisers’ experiences after surviving a SCA. The obligations of everyday life in young survivors of SCA often imply a high work load and complex tasks, e.g. due to being in the beginning of their career or even still studying. Healthcare personnel, as well as the society, need to acknowledge that although lucky to be alive and apparently well-functioning, young survivors of SCA may have persistent challenges that cause frustration and reduced quality of life.

**Supplementary Information:**

The online version contains supplementary material available at 10.1186/s12913-022-08674-z.

## Background

Sudden cardiac arrest (SCA) affects 150,000 to 450,000 individuals per year in the United States alone [1]. Over the past decade, survival after resuscitation from SCA has increased [2, 3]. Physical, cognitive, and emotional effects of surviving may be present for months or years after the SCA [4].

Studies have explored different aspects of surviving a SCA. A systematic review assessed specific domains of neurocognitive impairment following out-of-hospital cardiac arrest (OHCA), and showed that memory impairments were most frequently found, followed by impairments of executive functions and attention/information processing. In addition, general neurocognitive ability, motor function, language, and visuo-perceptual measures could be impaired [5]. Ørbo et al. [6] investigated cognitive recovery from 3 to 12 months after resuscitation and the associations between cognitive performance at 3 months and health-related quality of life (HRQL), psychological distress and work status after 12 months (*n* = 33). They found that memory impairments were the most common symptom, with minor improvements in cognitive performance from 3 to 12 months. Moreover, depressive symptoms increased and mental HRQL was reduced from 3 to 12 months. Better cognitive results at 3 months were correlated with better HRQL and return to work at 12 months. Geri et al. [7] interviewed survivors of OHCA 50 months after cardiac arrest and found that HRQL depended on the cerebral performance category (CPC) at hospital discharge. Patients with complete neurological recovery did not differ in HRQL compared to the general population, but in patients with incomplete neurological recovery, deep alterations were noted in almost all dimensions of HRQL compared to the general population. However, a 2017 systematic review demonstrated that a measure of quality of life specific to SCA survivors is not available [8]. Buanes et al. [9] found that 29 percent of patients were cognitively impaired (*n* = 30), with impaired short-time memory and executive function. In addition, a significant reduction in quality of life was reported. A review of the existing literature on psychological outcomes after SCA [10] described incidence rates for depression (14 to 45%), anxiety (13 to 61%) and post-traumatic distress syndrome (19 to 27).

Haydon et al. [11] conducted a systematic review and meta-synthesis of the qualitative literature exploring the experiences and quality of life of survivors of a SCA. The findings highlight both the psychological and physical changes influencing survivors’ perceptions of their life after the SCA. Five themes were identified: multitude of contrasting feelings; disruption in the continuum of time; new reality and psychological challenges; changed body with new limitations; and confrontation with death. Hence, surviving a SCA was seen as a major event for all involved, and what the survivor saw as a predictable future changed to the unknown. In previous qualitative studies, SCA survivors have underlined the importance of both psychologic and physical recovery, the impact of return to work or changes in work identity and the necessity of support from family members in the recovery process [12]. Moreover, they have reported a disrupted daily life from early on to several years after resuscitation [13, 14]. Mion et al. [15] investigated problems survivors of OHCA encountered. Results included fatigue, issues with memory/thinking, anxiety, loss of confidence, low mood and ongoing physical limitations. In addition, migraines, ICD related pain, loss of confidence when driving, pins and needles and general physical deconditioning were mentioned.

The body of evidence of effectiveness of rehabilitation interventions on cardiac arrest survivors is low [16]. A survey study in Sweden revealed that although local guidelines for follow-up exist at some hospitals, they are not uniformly applied or explicit [17]. Wagner et al. [18] suggest that attending a cardiac arrest rehabilitation programme may help SCA survivors towards a healthy transition to daily life after survival.

These earlier qualitative studies have included SCA survivors with an age ranging from 20 to 83 years [8, 10, 11, 13, 14, 18]. None of these have focused on young survivors solely, or on the participants’ physical condition prior to the SCA. Studies have shown a better prognosis when cardiac arrest occurs during or less than 60 min after exercise [19, 20]. However, to our knowledge, no studies have explored young exercisers’ experiences with the SCA. We assume that younger patients may have other needs and challenges than older patients, and also that life after the SCA may vary based on the individuals’ physical condition. Consequently, the aim of this study was to explore the aftermath of surviving a SCA in young exercisers.

## Methods

The study had a qualitative design, conducting in-depth individual interviews with young exercisers who had experienced a SCA. Findings on interpretation of warning signs and experiences with the healthcare system before, during and after the SCA have been published elsewhere [21]. The study adheres to the Consolidated criteria for reporting qualitative studies (COREQ) [22].

### Setting and participants

In Norway, the incidence of out-of-hospital cardiac arrest has been estimated to approximately 78 per 100.000 inhabitants per year [23]. The study presented here is part of a larger project aiming to explore incidence, risk factors, aetiology and prognosis for people aged 12 to 50 years who suffered SCA or sudden cardiac death in Norway in the period 2015–2017 (ethics approval number 2016/671 and 17/18457). Respondents were asked to be contacted for an interview for this study. Inclusion criteria were: Cardiac arrest of presumed cardiac cause during or less than 60 min after exercise, and/or persons who self-reported to exercise ≥ 5 h/week. We used a purposeful sampling strategy, inviting respondents accepting to be contacted with the highest reported level of exercising per week first.

### Data collection

To explore SCA survivors’ experiences we conducted in-depth, semi-structured interviews. During the process of developing an interview guide, all authors were involved in discussions. In addition, four patient representatives were consulted. Due to the Covid-19 pandemic and restrictions regarding travelling and physical meetings, interviews were conducted as video calls, using the digital software Microsoft Teams ©. Studies indicate that there are few differences between in-person and digital interviews [24, 25]. The use of video calls allowed us to include participants regardless of geographical location. Participants were asked to download an app beforehand and have access to microphone and camera. Video was not recorded, but the audio was taped and transcribed verbatim by an external transcriber, who had signed a non-disclosure agreement.

The interviews were performed by either the first (registered nurse, ph.d.), second (nurse anaesthetist, professor) or last author (family medicine doctor, associated professor), all females. In most interviews two interviewers were present. One of the interviewers had a leading role, providing the second interviewer the possibility to ask follow-up questions, if required. A semi-structured interview guide was used. The guide was based on non-structured interviews with prior survivors and their relatives, existing literature [8, 10, 11, 14], and developed over several iterations and discussions among the authors (Supplement 1). Throughout, the participants’ statements were mirrored, with the interviewer asking for correction or approval to verify that the participant had been interpreted correctly.

A method of reflexivity was applied throughout the data collection, as well as during the analysis [26, 27]. Before each interview, the leading interviewer wrote down own impressions, positioning and emotional investments based on previous interviews. After each interview, both interviewers wrote down initial impressions and thoughts from the interview, including non-verbal utterances from the participant.

Interviews were continued until saturation, indicated by data replication and the identification of no new themes [28], was reached.

### Data analysis

The data were analysed using systematic text condensation in-line with recommendations from Malterud [29]. The software program Hyper Research©, was used to facilitate the analysis process. The analysis followed four steps;

The first step was conducted by each of the authors respectively, and included reading the transcripts to familiarise with and get an overall impression of the data. In step two the first and second author (CH, ACLL) together identified meaning units in each transcript respectively, representing different aspects of the participants experiences after the SCA. This also allowed for discussions throughout, which lead to the identification of three preliminary themes across all transcripts. In step three, the meaning units were coded from these preliminary themes into different code groups. Here, the code groups were discussed with the last author (HMB) until consensus was reached. In the coding the focus was on experiences in relation to “being part of my surroundings”, “expecting normality but facing a new reality” and “lucky to be alive!”. Here we also identified illustrative quotations. Moreover, the reflexivity notes were reviewed to avoid interpretations based on the researchers’ own impressions and positionings. In step four, the second author synthesised the condensates from each code group, and presented a description of each category relating to the aim of the study. Table 1 gives an example of the analysis process.

**Table 1:** Example of the analysis process

**Meaning unit**	**Code group**	**Category**
My wife and I went to see a couples’ therapist, because we have very different reactions to the incident	Being part of my surroundings	Establishing a new everyday life
Early on, it was indicated that I wouldn’t be able to continue exercising, which was my primary leisure activity	Expecting normality but facing a new reality	
I was extremely lucky because the cardiac arrest occurred when I was at the gym	Lucky to be alive!	

### Ethical considerations

Due to the serious nature of the event leading to inclusion in the study, we gave participants an opportunity to contact either the interviewer or the last author if any negative feelings or further questions should appear after the interview. The study followed the guidelines in the Declaration of Helsinki [30], and was based on informed, willing and written consent to participate, as well as the right to withdraw from the study without any negative consequences. Principles for anonymity and confidentiality were followed. The study was approved by the Regional ethics committee (Ref. no 2016/671) and the local data protection authority at Oslo University Hospital (Ref. no 17/18457).

## Results

**Fig. 1:**
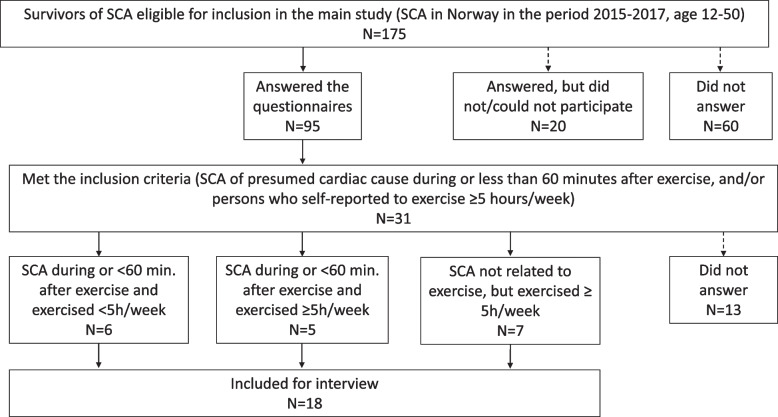
Inclusion

A total of 18 of 31 invited participants were included in the study. Inclusion is described in Fig. 1. Fourteen of the participants were male. Ten had experienced SCA during or less than 60 min after exercise, and eight were exercising ≥ 5 h per week prior to the incident. Their age ranged from 19–49 (median age 43) years at time of the SCA, in the following age categories: 19–24 years: four participants, 25–40 years: four participants and 40–50 years: ten participants. The time since the SCA ranged from 2.8 to 5.7 years. The interviews were conducted in the period September to December 2020, and lasted from 24 to 70 min. The thirteen participants in the main study that did not respond to the interview invitation had similar background demographics; ten were male, seven had experienced SCA during or less than 60 min after exercise, and four reported exercising ≥ 5 h per week. Age range 16–49 (median age 46), and the time since the SCA ranged from 3.4 to 5.8 years.

Through analysis we identified one main category relating to the aftermath of the SCA, namely ‘Establishing a new everyday life’, with related subgroups a) being part of my surroundings, b) expecting normality but facing a new reality and c) lucky to be alive!

## Establishing a new everyday life

All of the participants described that the SCA had changed their lives in one or another way. First, this was related to their interpersonal relations, both within family and among friends, and also in relation to their studies or work. Second, participants experienced various physical, cognitive and emotional symptoms that had not been there before the SCA. Third, all of the participants in some way expressed a gratitude for being alive, regardless of the abovementioned changes.

### Being part of my social context

The participants focused on the impact of the cardiac arrest upon their social context, both at the time of occurrence and after the incident. The social context included relatives and friends, as well as colleagues and the school or work situation. Many of the participants expressed that the cardiac arrest experience had been roughest on their relatives, especially among those who were present at the time of the cardiac arrest. Several of the relatives had performed the cardiopulmonary resuscitation and unlike the survivors of SCA who were unconscious, they have memories and experiences from throughout the course of the event. For those with small children, the event had been extremely challenging balancing a worry for their spouse and the future, alongside the responsibility for the children. One of the participants prompted:

«I realized the gravity of it, because everybody around me were so scared».

Some of the participants reported that the relatives were more anxious for them being too physically active, running alone in the woods or climbing in the mountains, but few reported of a daily worry. In addition, it seemed like the impact of the event weakened over time. One of the participants said:

«In the beginning, relatives and friends treated me differently, but now I think they have forgotten all about it»,

All of the participants emphasized a need to get back to their studies or work as soon as possible. One of them said: «It felt like working was more important than my life and health». 

There were great variations in when they had started studying or working after the cardiac arrest, from two weeks to three years. This did not seem to be related to the extent of physical sequels. In addition, there were great variations in whether the work had been adjusted to the participants’ needs. Statements varied from «My employer does not care how I am» or «It has been an extra challenge to be frozen out by my employer» to «the work was adjusted so that I worked what I managed» and stories about employers paying for psychotherapy. Several of the participants reported that they were forced to change their job, because the circumstances did not allow employees with an implantable cardioverter defibrillator (ICD), and that this felt frustrating.

### Expecting normality but facing a new reality

There were great variations in the extent of physical and cognitive symptoms reported after the cardiac arrest. Many of the participants described a reduced physical capacity, reduced short-time memory, difficulties with learning new information, being more emotional, being more sensitive to noises and having an instable temper. In addition, some of the participants reported of low pulse, fainting tendencies, and muscle spasms, which they related to the medication. Going from being relatively young, healthy and fit, this had great impact on their path to getting back to normal. One of the participants said:

«When I was discharged I was told that everything was okay. Someone should have told me that this was going to take time».

Still, some of the participants expressed no such symptoms after the cardiac arrest incident.

Since all of the participants had a high level of physical activity before the cardiac arrest, all of them were very concerned with when they could get back to their normal activities. One of the participants sighed:

«They told me that I could not do this, and could not do that, a long list of things I could not do that I loved doing».

Most of the participants had acquired a pulse-watch after the cardiac arrest, which they used to follow the pulse during activity. Still, none of them had a clear answer to how high or low the pulse could go before they should stop training or should worry. None of the participants had received clear instructions on how much they could work out, for how long or other limitations. This was something that several of the participants wished for, especially in cases where an arrythmia had caused the cardiac arrest, and participants being uncertain about what could trigger a new arrythmia. One of the participants prompted:

«I would like my physicians to tell me what my limitations are. I know that they are there, but I do not know what they are».

The fact that the participants were physically active and supposedly in good shape before the cardiac arrest, was prompted by most of the participants as being essential for their recovery after the event. 

There were great variations in how much the participants thought about the cardiac arrest in retrospect. Some participants cried throughout the interview and reported being depressed after the cardiac arrest. One felt that the ICD had led to too large difficulties in life because of necessary changes in work situation, and claimed that if he had had a choice, he would have declined the ICD- even though retrospectively knowing it had given him a shock twice. Others reported to be angry for having the cardiac arrest, while others again reported not thinking so much about it. One of the participants said:

«Cardiac arrest is something that happens to everyone else, not me, and something you watch on television».

Many of the participants described having a cardiac arrest as a shocking, scary, and a life-changing experience, and some worried for it to happen again. One of the participants stated:

«Before the cardiac arrest, my focus was on training and work and education. I have gotten more out of life and existence after the arrest due to filling the time with more important things».

### Lucky to be alive!

Even though the cardiac arrest was a traumatizing experience, most participants talked about how lucky they felt to survive. This was often related to location of the arrest, for example nearby a hospital, nearby an accessible defibrillator or close to other people. Many of the participants had wondered about what would have happened if they had been alone, running in the woods, «if my son had been in his room as usual» or «if we had been in our cabin”.

Further, it felt like this obliged them not to complain about the health care services they received, regardless of the quality, because they just had to be grateful to be alive. For example, one of the participants expressed:

«I would like to have some more information, but I am really very grateful for being here, and that we have a system that works»

The feeling of being lucky affected their views on life, and one of the participants stated:

«I get less annoyed over trifles after the cardiac arrest, because I really should not be here at all»

Still, this was a multicolored picture. One of the participants stated:

«Others say that I am lucky to be alive, but this is not black and white, and I have been angry a lot.»

The impression that «others» meant the participant should be grateful for being alive, and therefore mostly positive, was supported by several of the participants. One of them prompted:

«Maybe I should have been more grateful. But I have thanked those who should be thanked, and I must be allowed to still complain about an expensive soda even though I could have been dead.»

## Discussion

This is the first study exploring young exercisers’ experiences with the aftermath of a SCA. Being a young exerciser, and then returning to life after a SCA forced participants to get used to a new everyday life. They all acknowledged being part of surroundings that also were impacted by the event in one or another way. Moreover, many of the participants experienced both physical and cognitive impairment and emotional instability. Feeling lucky to be alive, most of the participants found it hard to complain about these changes.

Participants with small children reported of a need to balance a worry for their spouse, a worry about the future, alongside the responsibility for the children. No previous studies have explicitly explored young SCA survivors’ experiences in the aftermath of the event, so this is new knowledge. However, studies exploring the experiences of SCA survivors’ in general have been conducted. E.g. Forslund et al. [31] found that supportive social relationships affect health outcomes and are important for adjustment to illness. Survivors of SCA have also reported of changes in relationships with loved ones who are now in a caregiver role [32, 33]. This is in contrast to findings in our study, indicating that survivors felt responsible for their surroundings.

Survivors and their family members describe complex recovery journeys characterised by a range of psychosocial adjustment challenges [14]. A study from 2000 found that SCA survivors may have concerns about role changes in relationships and family, difficulty in being alone, and worries about resuming intimacy and sex [34]. These issues were not explored specifically nor spontaneously mentioned by the participants in our study. However, the young exercisers in this study might be more likely to return to most activities of daily living than other SCA populations.

Participants were eager to get back to their training, studies or work, and all but one had achieved this, even though three of the participants had been forced to change their job due to the nature of their work. We have not identified any qualitative studies underlining these findings.

Already in 2005, Lundgren-Nilsson et al. [35] underlined that we have limited understanding of specific barriers to community reintegration for patients and their families after experiencing a SCA. They stated that long-term reassessment is vital for societal reintegration of the SCA survivor after discharge from the hospital and rehabilitation. However, SCA survivors rarely receive information on options for support for themselves and their relatives [4]. Hence, the current system of care falls short by failing to organise discharge planning and long-term rehabilitation care resources, which, for many patients and their families, may be essential to maintain quality of life after SCA [18].

Several studies have reported changes in day-to-day functioning for survivors of SCA [13, 14, 32, 33]. Many of the participants in our study seemed surprised that life had not returned to normal. They reported of having various symptoms such as low pulse, fainting tendencies and muscle spasms, reduced short-time memory, learning difficulties, being more emotional, being more sensitive to noises and having an instable temper. Several studies have identified similar symptoms as experienced by the SCA survivors in our study [32, 36–39]. Studies have also identified high levels of anxiety and depression in SCA survivors after hospitalization [10, 14]; clinically significant depression has been reported in 8 to 45%, anxiety in 13 to 42%, and posttraumatic stress disorder (PTSD) in 19 to 27% of survivors [40–42]. Such symptoms seemed limited in our participants, even though they occurred.

The American Heart Association (AHA) scientific statement declares that patients and family members may lack awareness of cognitive deficits until the patient is discharged home, after which caregivers may be the first to recognize new cognitive challenges [4]**.** Ketilsdottir et al. [37] emphasised that support after hospital discharge for SCA survivors and relatives needs to be organised in a more structured fashion. Dainty et al. [36] support this, stating that there is a clear need for a more patient-centred outcome set for this population, and that psychologic assessment, return to work status and family input are key domains to be considered. This is supported by the findings in our study.

The European Resuscitation Council and European Society of Intensive Care Medicine guidelines 2021 for post-resuscitation care recommend for a multidisciplinary approach to rehabilitation for post–cardiac arrest patients. They also recommend the systematic organisation of follow-up care within 3-months of hospital discharge, including screening for cognitive and emotional impairments and fatigue, as well as providing information and support for survivors and relatives [43]. Findings in our study not only support this, but also underline the importance of individualising such services due to the heterogeneity of the population of young exercisers. Our participants reported great variations in both the extent of physical and cognitive symptoms after the cardiac arrest, and also variations in the emotional burden experienced in the aftermath of the SCA. Hence, we recommend that follow-up strategies in this population include thorough examination of physical, cognitive and emotional consequences of the SCA and that further follow-up are customized to the needs of each individual. One piece of information especially important to this population, yet missing in all participants in our study, was specific recommendations about exercise following the SCA.

All of the participants in our study in one way or another expressed a gratitude for being alive. This is supported by findings in several studies [11, 32, 33, 44, 45] also indicating that SCA survivors also have heightened appreciation for life. However, our participants also reported a frustration not feeling enabled to complain or to express negative feelings either related to the SCA experience or other events in their lives.

### Strengths and limitations

The study was conducted in a Norwegian population, and had a qualitative design, which limits the opportunity to generalise our findings. However, participants were of both genders, from different geographical locations nationwide, had various experiences with when and where the SCA occurred, and interviews were conducted until saturation was reached. Participants were all exercisers < 50 years of age, and had a presumed cardiac cause of SCA. Collated, this support the external validity of our findings. This was a self-selected group, and it is a limitation that we do not have information about why non-respondents chose not to participate in the study. However, the demographic background of respondents and non-respondents were similar.

To further increase the internal validity, we could have piloted the interview guide, or provided a member-check allowing participants to read through the transcripts and/or analysis. Due to confidentiality issues, and severity and sensitivity of the issues, we chose not to do this. The analysis process has been rigorously presented, and we used a method of reflexivity to keep our own impressions apart from the interpretation of the data. Moreover, codes and subgroups were discussed between all authors until consensus was reached. This support the trustworthiness of our findings.

## Conclusion

This study adds knowledge about young exercisers’ experiences after surviving a SCA. Information from health care personnel felt insufficient on what to expect when returning to everyday life, and an additional burden were added from concerned relatives. Young individuals tended to have large commitments in several areas of life, and the gap between their everyday work load prior to SCA and their capacity post SCA felt overwhelming. Healthcare personnel, as well as the society need to acknowledge that even apparently well-functioning survivors of a SCA could have persistent challenges that may cause frustration and reduced quality of life. Also, the emotional conflict of feeling lucky to be alive and at the same time be allowed to feel frustrated for small everyday struggles should be recognised.

### Implications for practice

The need to establish adequate and individualised follow-up services for SCA survivors and relatives after hospitalisation and rehabilitation is still present. Healthcare personnel have a responsibility to acknowledge the heterogeneity in a population of young exercisers and individualise follow-up accordingly. This population is particularly in need of information about exercise following the SCA, as well as information about potential challenges faced in the aftermath of a SCA.

## Supplementary Information

**Additional file 1. **

## Data Availability

The datasets generated and/or analysed during the current study are not publicly available due to confidentiality issues.

## Abbreviations

SCA: Sudden cardiac arrest
OHCA: Out-of-hospital cardiac arrest
HRQL: Health-related quality of life
ICD: Implantable cardioverter defibrillator
